# Effects of C2-Ceramide and Oltipraz on Hepatocyte Nuclear Factor-1 and Glutathione S-Transferase A1 in Acetaminophen-Mediated Acute Mice Liver Injury

**DOI:** 10.3389/fphar.2018.01009

**Published:** 2018-09-11

**Authors:** Xin Ma, Yicong Chang, Yuanyuan Zhang, Ishfaq Muhammad, Chenxi Shi, Rui Li, Changwen Li, Zhi Li, Yuexia Lin, Qing Han, Fangping Liu

**Affiliations:** ^1^College of Veterinary Medicine, Northeast Agricultural University, Harbin, China; ^2^Harbin Veterinary Research Institute, Chinese Academy of Agricultural Sciences, Harbin, China; ^3^Heilongjiang Key Laboratory for Animal Disease Control and Pharmaceutical Development, Harbin, China

**Keywords:** GSTA1, HNF-1, C2-ceramide, oltipraz, acetaminophen, liver injury

## Abstract

In this study, acetaminophen (APAP)-induced acute liver injury mice model was used to investigate the effects of C2-ceramide and oltipraz on hepatocyte nuclear factor 1 (HNF-1) and glutathione S-transferase A1 (GSTA1). Notably, C2-ceramide caused alteration in mice serum transaminases and liver tissue indexes, and aggravated hepatic injury, while oltipraz alleviated hepatic injury. By screening, the optimal concentrations of C2-ceramide and oltipraz were confirmed to be 120 and 150 μmol/L, respectively. In histopathology, karyolysis and more necrotic cells and bleeding spots were appeared on administration of C2-ceramide, but only a small amount of inflammatory cells infiltration was seen after oltipraz treatment. In addition, RT-PCR and western blot results revealed that the mRNA and protein expression levels of HNF-1 and GSTA1 in liver were significantly decreased (*p* < 0.01) with the administration of 120 μmol/L C2-ceramide. Meanwhile, GSTA1 content in serum increased up to 1.27-fold. In contrast, 150 μmol/L oltipraz incorporation to APAP model mice resulted in obvious elevation (*p* < 0.01) in the mRNA and protein expression levels of HNF-1 and GSTA1 in liver, and serum GSTA1 content decreased up to 0.77-fold. In conclusion, C2-ceramide could down-regulate the expression of HNF-1 and GSTA1 which exacerbated hepatic injury, while oltipraz could up-regulate the expression of HNF-1 and GSTA1 which mitigated hepatic injury.

## Introduction

Drug-induced liver injury (DILI) is associated with oxidative stress induced via the drug itself and/or its metabolites or the body’s allergic reaction to certain drugs (Kaplowitz, 2004). DILI is a key issue in drug development and clinical drug safety, which can be manifested as acute, chronic liver disease, and liver dysfunction (Kaplowitz, 2004). Due to lack of specificity, it is not easy to perform clinical diagnosis and treatment (Reuben et al., 2010). Studies reported that drug induced hepatotoxicity and the injury mechanisms bewitch the interest of scientists due to the increasing number of incidence of liver damage (Ilavenil et al., 2016; Cheng et al., 2017; Kim et al., 2017). Acute liver injury is frequently related with increased enzyme activity without clinical symptoms and results in acute fulminant hepatic failure (Michaut et al., 2016). The acute liver injury model induced by acetaminophen (APAP) is used as the most typical DILI model in clinical trials. In rodents, especially in mice, it is common to study APAP induced liver injury model *in vivo* (Yuan et al., 2010). Currently, the evaluation of liver injury relies on the determination of various indicators in serum (aminotransferases) (Ozer et al., 2008) and in liver tissues (SOD, MDA, GSH, etc) (Hsieh et al., 2015). Previously, GSTA1 was identified as a more sensitive indicator than aminotransferases by using APAP-induced hepatic injury (Liu et al., 2014).

Glutathione S-transferase A1, a key phase II enzyme involved in the detoxification reactions (Iorio et al., 2014), is one of the important members of GST family (Ma et al., 2017), which is generally believed as a key factor in detoxification pathways. GSTA1 is an important component of antioxidant defense system, which can catalyze a number of exogenous chemicals such as carcinogens, environmental toxins, certain drugs combined with GSH, promote their degradation in the intracellular clearance and protect body systems (Board and Menon, 2013; Liu et al., 2016). GSTA1 has non-selenium-dependent GSH-Px activity, scavenge lipid free radicals and oxidative stress reaction products, and contains the function of anti-lipid peroxidation (Singhal et al., 2015). GSTA1 is released into serum from liver for detoxification on the onset of DILI (Shi et al., 2017). Meanwhile, GSTA1 can activate some molecular mechanisms, regulate the signal transduction and prevent cell apoptosis (Adnan et al., 2012). The detoxification of GSTA1 to extraneous materials and reactive oxygen species may represent an important cellular reaction process in oxidative stress.

Hepatocyte nuclear factor (HNF) is a type of transcription factor that regulates gene expression in the liver, contains an evolutionarily conserved DNA-binding domain, regulates the expression of the target genes by binding to *cis*-acting elements, and plays an important role in the differentiation and metabolism of hepatocytes at transcriptional level (Hayashi et al., 1999). HNF-1 has two forms, namely HNF-1 and variant HNF-1 (vHNF-1) (Pontoglio, 2000). HNF-1 accounts for 95% of the total HNF1-like protein in liver, while vHNF-1 expression is very low (Pontoglio, 2000). HNF-1 plays an important role in promoting the development of liver and maintaining the biological function of hepatocytes, also controls the expression of liver specific genes (Boudreau et al., 2001; Michels and Hagen, 2009).

Ceramide is an endogenous bioactive lipid second messenger and a member of sphingolipid family that plays a key role in mediating cell proliferation, differentiation, migration, senescence, and apoptosis (Dany and Ogretmen, 2015; Li and Zhang, 2015). Furthermore, ceramide exhibits significant combination therapy efficacy when used in combination with other drugs due to its involvement in various tumor suppressor signaling pathways including apoptosis, autophagy, and metastasis (Thayyullathil et al., 2014; Li and Zhang, 2015). C2 is a synthetic short-chain ceramide compound with endogenous ceramide activity (Zhu et al., 2014). Oltipraz is a kind of extract in cruciferous vegetables with anti-cancer effect (Langouet et al., 1995). Oltipraz has been extensively studied as a cancer chemopreventive agent, and it is also used for the treatment of liver cirrhosis (Kang et al., 2002, 2003). Kang et al. (2002) have shown that oltipraz can increase the survival rate of liver cirrhosis in rats through improvement of liver function, regeneration of liver cirrhosis and reduction of liver fibrosis. Therefore, the present study aimed to investigate the effects of C2 and oltipraz on HNF-1 and GSTA1 expression in APAP-induced acute hepatic injury to clarify the roles of HNF-1 and GSTA1 in liver injury.

## Materials and Methods

### Reagents

APAP was purchased from Yong An Chemical Industry (Anhui, China). C2 and Oltipraz were purchased from Sigma-Aldrich Trading Co., Ltd. (Shanghai, China). The detection kits of ALT, AST, SOD, MDA, GSH and GSH-Px were obtained from the Nanjing Jiancheng Institute of Biotechnology (Nanjing, China). The GSTA1 detection kit was obtained from American RapidBio (RB) company. TRIzol reagent was provided by Life Technologies Corporation (Cal., United States). Reverse Transcription kit was purchased from Takara Corp (Dalian, China). TransStart Top Green qPCR SuperMix kit was obtained from TransGen Biotech Co., Ltd. (Beijing, China). Polyclonal antibodies of GSTA1 and HNF-1 were bought from ABclonal Biotechnology Co., Ltd. (Boston, MA, United States).

### Animals and Treatment

Adult male Kunming mice (8-week-old, 18–22 g) were obtained from the Harbin Pharmaceutical Group Co. Ltd. General Pharm. Factory, laboratory animal centre. The animals were housed in a controlled environment under standard conditions at a temperature (20 ± 2°C), 12 h light/dark cycle, a relative humidity (40–60%), and allowed free access to food (standard mice pellets), water and were acclimatized for at least 1 week before use. This study was carried out in accordance with the recommendations of the China National Institutes of Health Guidelines for the Care and Use of Laboratory Animals. The protocol was approved by the Harbin Veterinary Research Institute Animal Ethics Committee (approval number was SYXK (Hei) 2012-2067).

### Experimental Design

#### C2-Ceramide and Oltipraz Optimal Concentrations Screening

(1) Mice were randomly divided into five groups (*n* = 8), including control group, APAP (200 mg/kg) model group, C2 (100, 120, and 140 μmol/L) + APAP groups. (2) Mice were randomly divided into five groups (*n* = 8), including control group, APAP (200 mg/kg) model group, oltipraz (OL) (140, 150, and 160 μmol/L) + APAP groups. (3) Mice were randomly divided into seven groups (*n* = 8), including control group, C2 (100, 120, and 140 μmol/L) groups and oltipraz (140, 150, and 160 μmol/L) groups. C2 and oltipraz were dissolved in pure DMSO, and diluted in physiological saline to result in final concentrations. The doses corresponding to the concentrations of C2 (100, 120, and 140 μmol/L) are 342, 410, and 478 μg/kg, respectively. The doses corresponding to the concentrations of oltipraz (140, 150, and 160 μmol/L) are 317, 340, and 362 μg/kg, respectively. Ten milliliters solution was given to mice per kilogram of body weight. The control group received an equal volume of DMSO and physiological saline. After administration of different drugs for 12 h, mice were subsequently anesthetized with ether (Shenyang Chemical Reagent Factory) and sacrificed by cervical dislocation, following serum separation for the determination of ALT and AST.

#### Effects of C2-Ceramide and Oltipraz on APAP-Induced Liver Injury Model

The animals were randomly divided into six groups (*n* = 8), including control group, APAP model group, C2-ceramide group (C2), C2-ceramide + APAP group (C2+APAP), oltipraz group (OL) and oltipraz + APAP group (OL+APAP). C2 and C2+APAP groups were administered 120 μmol/L (410 μg/kg) C2, OL and OL+APAP groups were administered 150 μmol/L (340 μg/kg) oltipraz. Then APAP, C2+APAP and OL+APAP groups were administered 200 mg/kg APAP by gavage, and control group was given an equal volume of physiological saline and DMSO. After 12 h, mice were subsequently anesthetized with ether and sacrificed by cervical dislocation, following which serum was separated for the determination of ALT, AST and GSTA1. Then, the liver was isolated and stored at -80°C for analysis of other indicators, real-time RT-PCR and western blot, except for the left lobe, which was used for histopathological studies.

### Determination of Serum ALT and AST

Alanine aminotransferase and AST activities in serum were determined using detection kits according to the manufacturer’s instructions.

### Determination of MDA, SOD, GSH and GSH-Px in Liver

Malondialdehyde, SOD, GSH, and GSH-Px in liver were determined using detection kits according to the manufacturer’s instructions.

### Mice Liver Pathology

Formalin-fixed specimens were embedded in paraffin and sectioned for 5 μm thickness according to the routine procedure. After hematoxylin and eosin (H&E) staining, the slides were observed for conventional morphological evaluation under a light microscope and photographed at 400× magnification.

### Total RNA Isolation and RT-PCR Analysis

Total RNA was prepared from 50 to 100 mg of liver tissue using TRIzol reagent according to the manufacturer’s instructions. Isolated RNA (1 μg/20 μl reaction volume) was used for first-strand cDNA synthesis using Reverse Transcription Kit. The levels of mRNA expression were quantified by real-time RT-PCR with SYBR^®^Premix Ex Taq^TM^ II (Tli RNaseH Plus) and LightCycler^®^ 96 System. A real-time RT-PCR reaction was performed in a 20 μl volume containing 10 μl SYBR Premix Ex Taq^TM^ and 5 μM of each primer. Thermocycling was performed as follows: 94°C, 5 min for denaturation and then 40 cycles of PCR (94°C, 30 s; 58°C, 40 s; 72°C, 40 s) and 72°C, 5 min. The mRNA levels were normalized against β-actin mRNA. Primer sequences used for real-time RT-PCR are shown in **Table 1**.

**Table 1:** Primer sequences for real-time RT-PCR.

Names	Accession No.	Forward primer (5′ to 3′)	Reverse primer (5′ to 3′)
HNF-1	NM_009327.3	CCCTGACAACTTCCTTCCTG	GTGGCTTCTCTCTGCTGGTC
GSTA1	NM_008181.3	TGGGAATTTGATGTTTGACC	CAGGGCTCTCTCCTTCATGT
β-actin	NM_007393.3	AGCGTCCTGGTCTTGATGTCTGT	GAGGTCCCAGGTAGATGGTGAAT

### Western Blot Analysis

Total protein was extracted from 20 mg of liver tissue using 200 μl lysis solution according to the manufacturer’s instructions. Protein concentrations were measured by using the NANODROP 2000c Spectrophotometer to keep all sample protein concentrations consistent. After SDS polyacrylamide gel electrophoresis, protein was transferred onto NC membrane by transmembrane. The NC membrane was incubated with primary antibody overnight, and then incubated with secondary antibody for 1 h. ECL droplets were deposited on the NC membrane and the NC membrane was placed in the Tanon 5200 system to develop. Finally, the expression of HNF-1 and GSTA1 protein was analyzed by Image J software.

### Determination of GSTA1 Content in Liver

Glutathione S-transferase A1 concentration in serum was detected with a Mouse GSTA1 ELISA Kit that used Purified Mouse GSTA1 to coat microtiter plate wells, making a solid-phase antibody. The procedure was performed according to the manufacturer’s instructions.

### Statistical Analysis

Values were expressed as the mean ± SD, and statistical significance was determined by one-way analysis of variance using the SPSS software 19.0. The Tukey’s multiple comparison test in *post hoc* Multiple Comparisons was used to examine statistical significance (*p* < 0.05 and *p* < 0.01) between groups.

## Results

### Screening for Optimal C2-Ceramide and Oltipraz Concentrations

**FIGURE 1:**
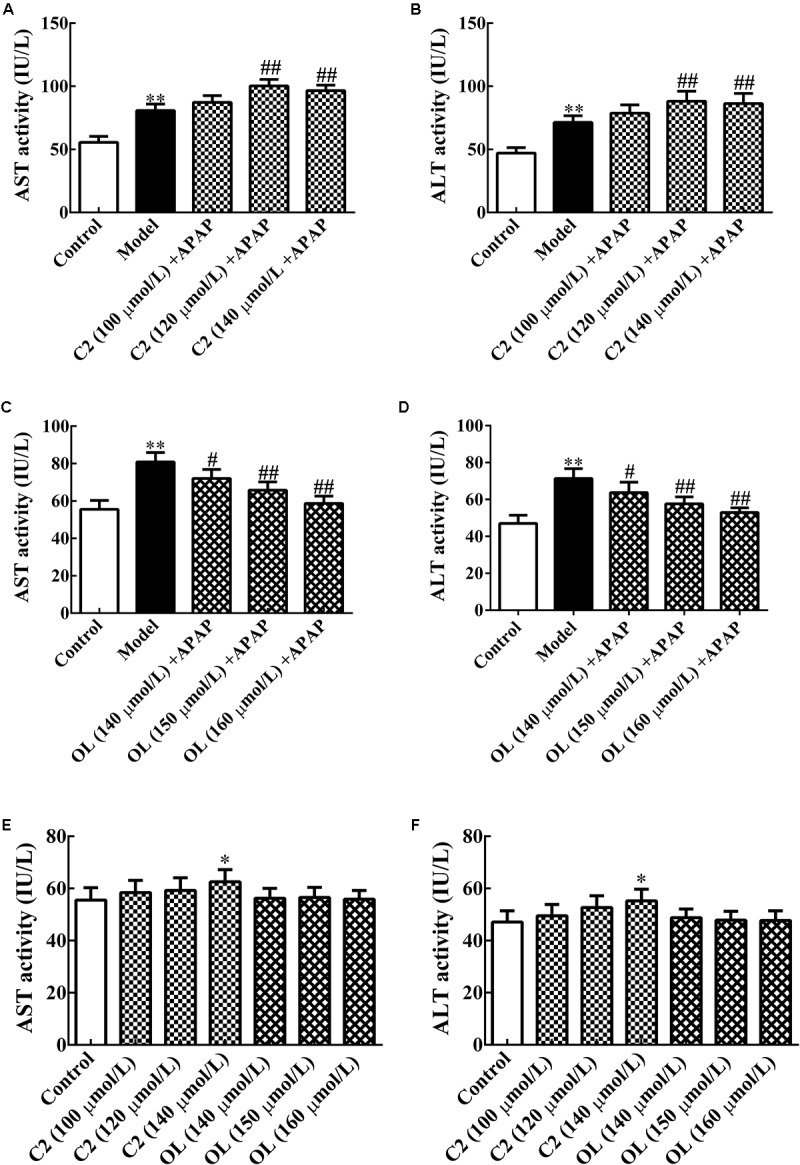
**(A,B)** Effects of different concentrations of C2-ceramide on AST and ALT activities in APAP-induced hepatic injury. **(C,D)** Effects of different concentrations of oltipraz on AST and ALT activities in APAP-induced hepatic injury. **(E,F)** Effects of different concentrations of C2-ceramide and oltipraz on AST and ALT activities in normal mice. Values are expressed as the mean ± SD for each group (*n* = 8). ^∗^*p* < 0.05, ^∗∗^*p* < 0.01 compared with control group; ^#^*p* < 0.05, ^##^*p* < 0.01 compared with model group.

The results of screening for optimal C2 and oltipraz concentrations were presented in **Figure 1**. Compared with control group, significant increase (*p* < 0.01) in serum AST and ALT activities were observed in model group. Compared to model group, serum AST and ALT activities were significantly increased (*p* < 0.01) with 120 and 140 μmol/L C2 concentration. Meanwhile, serum AST and ALT activities were obviously decreased (*p* < 0.05) with 140 μmol/L oltipraz concentration, and significantly decreased (*p* < 0.01) with 150and 160 μmol/L oltipraz concentration.

Compared with control group, it has been showed that serum AST and ALT activities were significantly increased (*p* < 0.05) with 140 μmol/L of C2 which indicated that the optimum concentration of C2 was chosen less than 140 μmol/L. Meanwhile, there were no obvious changes in other C2 groups and oltipraz groups. Thus, we chose 120 μmol/L for C2 and 150 μmol/L for oltipraz, which cannot affect normal mice to perform subsequent experiments.

### Effects of C2-Ceramide and Oltipraz on APAP-Induced Hepatic Injury

**FIGURE 2:**
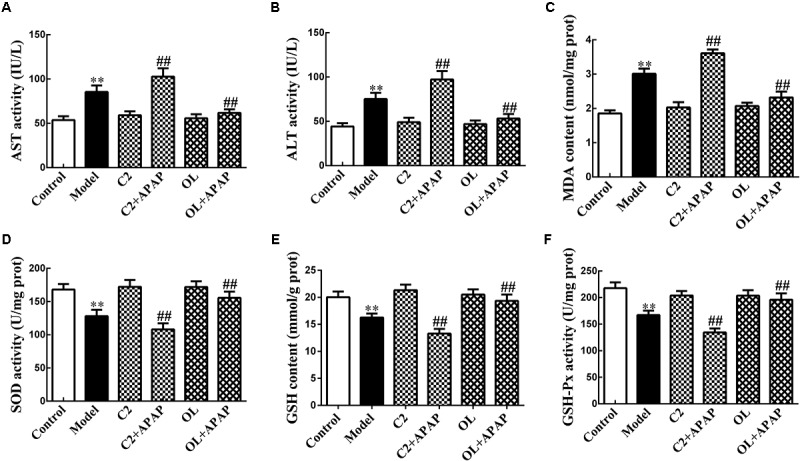
The effects of C2-ceramide and oltipraz on serum AST **(A)** and ALT **(B)** activities, liver MDA content **(C)**, SOD activity **(D)**, GSH content **(E),** and GSH-Px activity **(F)** in APAP-induced hepatic injury. Values are expressed as the mean ± SD for each group (*n* = 8). ^∗∗^*p* < 0.01 compared with control group; ^##^*p* < 0.01 compared with model group.

The results of transaminases in serum and hepatic oxidative stress indicators in liver were presented in **Figure 2**. Serum ALT and AST activities and liver MDA content were significantly increased (*p* < 0.01). Meanwhile, SOD and GSH-Px activities and GSH content in liver were obviously decreased (*p* < 0.01) in model group with respect to control group. Compared with model group, it has been noted that serum ALT and AST activities and liver MDA content were significantly increased (*p* < 0.01). Besides, liver SOD, GSH-Px, and GSH levels were markedly decreased (*p* < 0.01) in C2+APAP group. In contrast, serum AST and ALT activities and liver MDA content were significantly decreased (*p* < 0.01). Moreover, liver SOD, GSH-Px, and GSH levels were obviously increased (*p* < 0.01) in OL+APAP group.

### Effects of C2-Ceramide and Oltipraz on Liver Pathology of Mice

**FIGURE 3:**
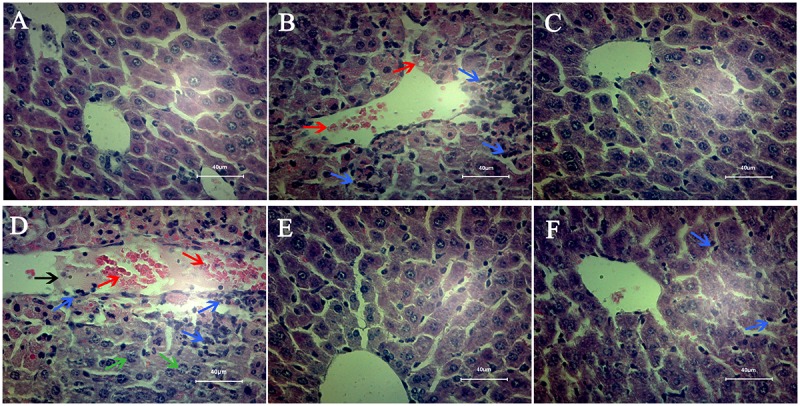
Mice liver pathology. Liver sections were stained by hematoxylin and eosin. All the liver sections were examined by light microscopy and the images were displayed at 400× the original magnification. **(A)** Control group; **(B)** model group; **(C)** C2-ceramide group; **(D)** C2-ceramide + APAP group; **(E)** Oltipraz group; **(F)** Oltipraz + APAP group. The bleeding was pointed out by the red arrows. The inflammatory cells infiltration was marked out by blue arrows. The karyolysis was pointed out by the green arrows. Black arrow meant that there is exudate in the blood vessels.

Pathological section results of each group were presented in **Figure 3**. In model group (**Figure 3B**), the hepatic cords were obscure and severely damaged, multiple inflammatory infiltrates and hemorrhages were found in the hepatocytes. The hepatocytes morphology and hepatic cords in C2 and OL groups (**Figures 3C,E**) were normal and clear as observed in control group (**Figure 3A**). More lesions appeared in the C2+APAP group (**Figure 3D**) in comparison to model group, which showed the blurred hepatic cord, serious bleeding, karyolysis, more necrotic cells and inflammatory cells infiltration. OL+APAP group (**Figure 3F**) was close to the control group, but there was still a small amount of inflammatory cells infiltration in liver tissues.

### Serum GSTA1 Content in APAP-Induced Hepatic Injury

**FIGURE 4:**
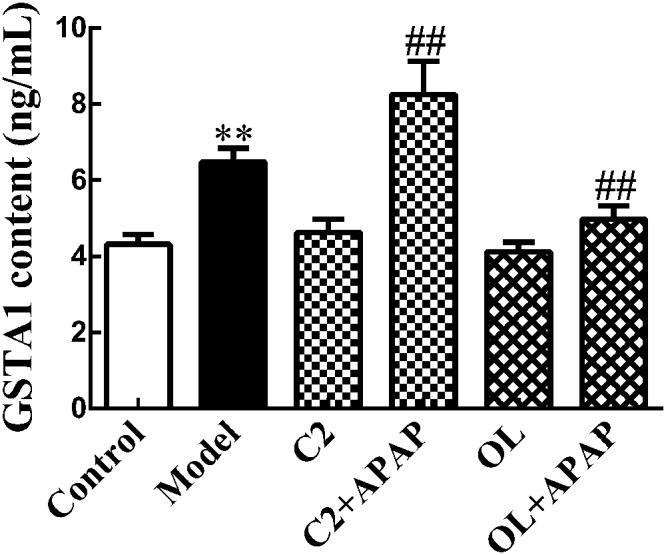
Effects of C2-ceramide and oltipraz on serum GSTA1 content in APAP-induced hepatic injury. Values are expressed as the mean ± SD for each group (*n* = 8). ^∗∗^*p* < 0.01 compared with control group; ^##^*p* < 0.01 compared with model group.

The changes of serum GSTA1 content in APAP-induced hepatic injury were presented in **Figure 4**. Compared with control group, a significant increase (*p* < 0.01) in serum GSTA1 content was observed in model group. Moreover, a significant increase (*p* < 0.01) in serum GSTA1 concentration was found in C2+APAP group compared to model group. In contrast, a significant decrease (*p* < 0.01) has been noted in serum GSTA1 content in OL+APAP group.

### HNF-1 and GSTA1 mRNA Expression in APAP-Induced Hepatic Injury

**FIGURE 5:**
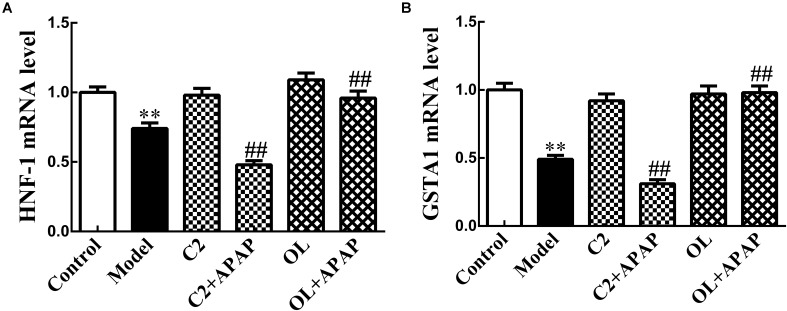
Effects of C2-ceramide and oltipraz on liver HNF-1 **(A)** and GSTA1 **(B)** mRNA expression in APAP-induced hepatic injury. Values are expressed as the mean ± SD for each group (*n* = 8). ^∗∗^*p* < 0.01 compared with control group; ^##^*p* < 0.01 compared with model group.

The results of HNF-1 and GSTA1 mRNA expression by Real-time RT-PCR were presented in **Figure 5** in the presence of C2 and oltipraz. Compared with control group, the expression of HNF-1 and GSTA1 mRNA in model group were significantly decreased (*p* < 0.01). Significant decrease (*p* < 0.01) in the expression of HNF-1 and GSTA1 mRNA were observed in C2+APAP group in comparison with model group. In contrast, the expression of HNF-1 and GSTA1 mRNA were significantly increased (*p* < 0.01) in OL+APAP group. Intriguingly, the expression levels of GSTA1 mRNA were consistent with the trend of HNF-1 in each group.

### HNF-1 and GSTA1 Protein Expression in APAP-Induced Hepatic Injury

**FIGURE 6:**
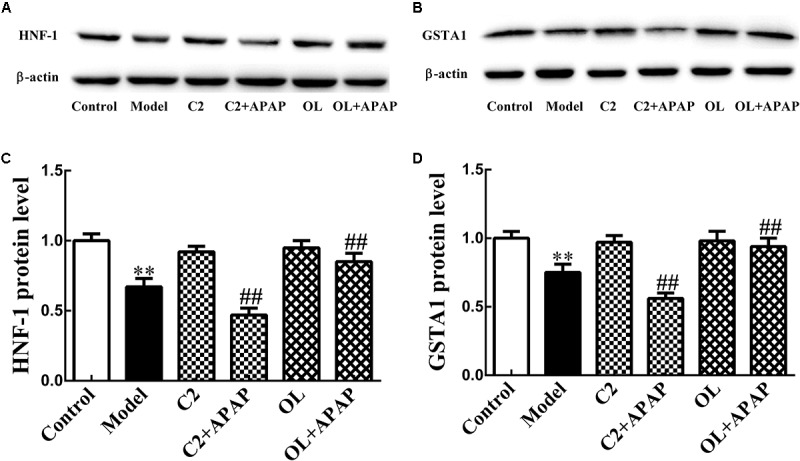
Original blots for HNF-1 **(A)** and GSTA1 **(B)**. Effects of C2-ceramide and oltipraz on liver HNF-1 **(C)** and GSTA1 **(D)** protein expression in APAP-induced hepatic injury. Values are expressed as the mean ± SD for each group (*n* = 8). ^∗∗^*p* < 0.01 compared with control group; ^##^*p* < 0.01 compared with model group.

The protein expression level of HNF-1 and GSTA1 were quantified in liver and displayed in **Figure 6**. Compared with control group, the expression of HNF-1 and GSTA1 protein were significantly decreased (*p* < 0.01) in model group. Compared to model group, the expression of HNF-1 and GSTA1 protein were significantly decreased (*p* < 0.01) in C2+APAP group. While, in OL+APAP group, HNF-1 and GSTA1 showed significant increase in protein expression (*p* < 0.01). Surprisingly, similar trends were found in GSTA1 and HNF-1 protein expression.

## Discussion

Previously, our research group proved that GSTA1 was a more sensitive and accurate indicator to represent liver injury than ALT *in vivo* and *in vitro* (Liu et al., 2014; Chang et al., 2017; Li et al., 2017). Lu et al. (2012) reported that GSTA1 was reduced in CCl_4_-induced acute liver injury and dioscin could protect liver with increased GSTA1 expression. Similarly, we investigated the hepatoprotective effects of *Folium Syringae* against APAP-induced hepatic injury *in vitro* and *in vivo*, and the results showed that *Folium Syringae* could protect the integrity of hepatocytes and thus reduce the release of liver GSTA1, which contributed to improve liver detoxification (Shi et al., 2017). HNF-1, a transcription factor located on chromosome 12, involved in the regulation of the expression of several liver-specific genes (Jawaid et al., 2016). Although HNF-1 is highly expressed in liver (Bjorkhaug et al., 2005), research about the role of HNF-1 in hepatic injury is insufficient. Therefore, the aim of our study was to investigate the effects of C2 and oltipraz on HNF-1 and GSTA1 expression and the roles of HNF-1 and GSTA1 in APAP-induced acute hepatic injury.

Ceramide is a central molecule in the biosynthetic pathway of sphingolipids and a potent tumor suppressor by regulating cell proliferation, differentiation, senescence, and apoptosis (Yabu et al., 2015). Jiang et al. (2017) have shown that the combination of C2 and sorafenib provides synergistic inhibition of hepatocellular carcinoma (HCC) cells through cell cycle arrest, ROS production, and caspase-dependent apoptosis. Moreover, C2 and sorafenib combination treatment increased ROS accumulation in Bel7402 cells and scavenging ROS by antioxidant tocopherol and lipoic acid could restore cell viability (Jiang et al., 2017). In general, phase II enzymes mediate the coupling reaction to help eliminate toxic materials. If the phase II response is insufficient, activated poisons may cause tissue damage, inflammation, genetic alterations, and/or inactivation of tumor suppressor genes (Choi et al., 2010). Oltipraz is a member of a novel class of dithiothreitol, many of which are capable of preventing cells from mitochondrial dysfunction induced by arachidonic acid (AA) + iron (Shin and Kim, 2009). Studies have shown that dithiothreitol, including oltipraz and some of its metabolites, induces transactivation of genes encoding phase II enzymes (Egner et al., 1994; Petzer et al., 2003). Moreover, oltipraz has an antioxidant effect at least in part by inducing phase II enzyme and/or Mn-SOD genes (Kwon et al., 2009). Oltipraz metabolites increase the cellular GSH content (Kwon et al., 2009). In addition, oltipraz can inhibit AA + iron-induced ROS production and its metabolites can abolish the ability of AA-induced mitochondrial superoxide production, thereby protecting cells from ROS-induced apoptosis (Kwon et al., 2009; Shin and Kim, 2009).

Firstly, we screened the optimum concentrations of optimal C2 and oltipraz. It has been found that AST and ALT activities were significantly increased (*p* < 0.01) in APAP-induced hepatic injury with 120 and 140 μmmol/L of C2, while these were obviously decreased (*p* < 0.05 or *p* < 0.01) with 140, 150, and 160 μmmol/L of oltipraz relative to model group. Meanwhile, 120 μmmol/L of C2 and all concentrations of oltipraz could not affect normal mice. Therefore, we chose 120 μmmol/L of C2 and 150 μmmol/L of oltipraz for subsequent experiments. Then, APAP-induced liver injury mice model was treated with C2 and oltipraz to study the alterations in liver indexes. Compared to model group, serum transaminases (ALT, AST) and liver MDA were obviously increased and liver SOD, GSH, and GSH-Px were significantly decreased in C2+APAP group. In contrast, serum AST and ALT and liver MDA were significantly reduced. While liver SOD, GSH, and GSH-Px were obviously enhanced in OL+APAP group. Similarly, the results of histopathological observation showed that there was less inflammatory cell infiltration in OL+APAP group, almost close to control group. While, more necrotic cells and karyolysis appeared in C2+APAP group relative to model group. Meanwhile, there were no obvious lesions and all indexes were not changed in C2 and OL groups. These findings suggested that C2 aggravated liver injury and oltipraz mitigated liver injury.

Transcription factor HNF-1 contains DNA binding regions that are evolutionarily conserved, regulates the expression of target genes through the combination of *cis*-acting elements and plays an important role in the regulation of hepatocyte differentiation and detoxification (Mosialou et al., 2011; David-Silva et al., 2013). In this study, the expression level of HNF-1 mRNA and protein were determined by real-time fluorescence quantitative analysis and western blot. It was found that the relative expression of HNF-1 mRNA and protein in model group were significantly lower than those in control group, which proved that APAP induced acute liver injury could reduce HNF-1 expression. The relative expression of HNF-1 mRNA and protein decreased obviously in C2+APAP group. In contrast, HNF-1 mRNA and protein level significantly increased in OL+APAP group relative to model group. While, compared to control group, there was no significant difference in C2 and OL groups. These results suggested that C2 could down-regulate the expression of HNF-1 and oltipraz could up-regulate the expression of HNF-1 in the presence of APAP-induced liver injury. Moreover, there was no effect on HNF-1 expression under the optimum concentrations of C2 and oltipraz. However, higher studies are needed to investigate the molecular mechanism behind the stimulation of HNF-1 expression with C2 and oltipraz treatment in different concentrations.

Glutathione S-transferase A1 is a key enzyme in the glutathione binding reaction and plays an important role in the metabolic process of APAP. GSTA1 can catalyze the reaction of nucleophilic glutathione with metabolites of APAP and degrade the toxic substances in the body (Bailey et al., 2012). The increased expression of GSTA1 can reduce the proliferation of cancer cells and protect cells against the damage of lipid peroxidation (Matic et al., 2013), which has been an important mechanism of anti-oxidative stress in detoxification of toxic substances and reactive oxygen species (Marcus et al., 2012; Sohn et al., 2013). In this study, the expression level of GSTA1 mRNA and protein in model group were significantly lower than those in the control group, and GSTA1 content in serum was obviously increased. Thus, it has been demonstrated that GSTA1 was released from liver to serum for detoxification in APAP-induced acute liver injury. There was no significant difference in C2 and OL groups in GSTA1 content relative to control group. Our data showed that the relative expression of GSTA1 mRNA and protein in C2+APAP group were obviously decreased and GSTA1 content in serum was significantly increased. While, contrasting results have been found in OL+APAP group. More importantly, these results were consistent with the trend of HNF-1 mRNA and protein expression. Therefore, there might be a certain intrinsic link between them and further molecular studies are needed to investigate the mechanism behind the regulation of transcription factor HNF-1 and its downstream gene GSTA1 in APAP-induced liver injury.

## Conclusion

C2 could aggravate liver damage and oltipraz could alleviate liver damage in APAP-induced acute hepatic injury in mice. Moreover, HNF-1 and GSTA1 expression could be down-regulated by C2 which exacerbated hepatic injury, while up-regulated by oltipraz which mitigated hepatic injury. Accordingly, HNF-1 and GSTA1 are involved in acute hepatic injury induced by APAP, and there might be a certain intrinsic molecular association between HNF-1 and GSTA1.

## Author Contributions

FL supervised the whole experiments. XM and YC performed the practical work and completed the experiments. YZ helped to analyzed the data for the work and revised the article. CS, RL, CL, ZL, YL, and QH provided help during the experiments. IM helped in improving language expression.

## Conflict of Interest Statement

The authors declare that the research was conducted in the absence of any commercial or financial relationships that could be construed as a potential conflict of interest.

## Abbreviations

ALT: alanine aminotransferase
APAP: N-acetyl-p-aminophenol/acetaminophen/paracetamol
AST: aspartate aminotransferase
C2: C2-ceramide
DILI: drug-induced liver injury
DMEM: dulbecco’s modified eagle medium
DMSO: dimethyl sulfoxide
GSH: glutathione
GSH-Px: glutathione peroxidase
GST: glutathione S-transferases
GSTA1: glutathione S-transferase A1
HNF-1: hepatocyte nuclear factor 1
MDA: malondialdehyde
NAPQI: N-acetyl-p-benzoquinone imine
OL: oltipraz
SOD: superoxide dismutase.
